# Transcranial versus Direct Cortical Stimulation for Motor-Evoked Potentials during Resection of Supratentorial Tumors under General Anesthesia (The TRANSEKT-Trial): Study Protocol for a Randomized Controlled Trial

**DOI:** 10.3390/biomedicines9101490

**Published:** 2021-10-16

**Authors:** Tammam Abboud, Thomas Asendorf, Jutta Heinrich, Katharina Faust, Sandro M. Krieg, Kathleen Seidel, Dorothee Mielke, Cordola Matthies, Florian Ringel, Veit Rohde, Andrea Szelényi

**Affiliations:** 1 Department of Neurosurgery, University Medical Center Göttingen, Robert-Koch-Straße 40, 37075 Göttingen, Germany; dorothee.mielke@med.uni-goettingen.de (D.M.); veit.rohde@med.uni-goettingen.de (V.R.); 2 Department of Medical Statistics, University Medical Center Göttingen, Humboldtallee 32, 37073 Göttingen, Germany; thomas.asendorf@med.uni-goettingen.de; 3 Clinical Trial Unit, University Medical Center Göttingen, Robert-Koch-Straße 40, 37075 Göttingen, Germany; Jutta.Heinrich@med.uni-goettingen.de; 4 Department of Neurosurgery, Charité University Clinic, 10117 Berlin, Germany; katharina.faust@charite.de; 5 Department of Neurosurgery, Klinikum Rechts der Isar, School of Medicine, Technische Universität, 81675 München, Germany; sandro.krieg@tum.de; 6 Department of Neurosurgery, Inselspital, University Hospital, 3010 Bern, Switzerland; kathleen.seidel@insel.ch; 7 Department of Neurosurgery, University Medical Center Würzburg, 97080 Würzburg, Germany; matthies_c@ukw.de; 8 Department of Neurosurgery, University Medical Center Mainz, 55131 Mainz, Germany; florian.ringel@unimedizin-mainz.de; 9 Department of Neurosurgery, Campus Grosshadern, Ludwig-Maximilians-University, 81377 Munich, Germany; Andrea.Szelenyi@med.uni-muenchen.de

**Keywords:** threshold criterion, amplitude criterion, intraoperative monitoring, transcranial motor-evoked potentials, direct cortical stimulation, threshold level

## Abstract

Background: Monitoring of motor function during surgery for supratentorial tumors under general anesthesia applies either transcranial electrical stimulation (TES) or direct cortical stimulation (DCS) to elicit motor-evoked potentials. To date, there is no guideline that favor one method over the other. Therefore, we designed this randomized study to compare between both methods regarding the prediction of postoperative motor deficits and extent of tumor resection. Methods: This is a multicenter (six centers in Germany and one in Switzerland), double blind, parallel group, exploratory, randomized controlled clinical trial. Patients without or with mild paresis, who are scheduled for surgical resection of motor-eloquent brain tumors under general anesthesia will be randomized to surgical resection under TES or surgical resection under DCS. The primary endpoint is sensitivity and specificity in prognosis of motor function 7 days after surgery. The main secondary endpoint is the extent of tumor resection. The study is planned to include 120 patients within 2 years. Discussion: The present exploratory study should compare TES and DCS regarding sensitivity and specificity in predicting postoperative motor deficit and extent of tumor resection to calculate the required number of patients in a confirmatory trial to test the superiority of one method over the other.

## 1. Introduction

Cancer of the central nervous system, with glioma being its most common histological type, was responsible for 721,787 disability adjusted life years (DALYs) in western Europe and 150,993 DALYs in Germany according to counts from 2016 [1]. Surgical resection is the treatment of choice for brain tumors in most cases. The goal is to achieve maximum tumor resection. This should be performed without or with only minimal postoperative neurological deficits in order to avoid relevant impairment of the quality of life of the patients after the operation, which can affect their ability to receive adjuvant therapy. Intraoperative monitoring is an established method to ensure the integrity of the corticospinal tract (CST) during surgical removal of brain tumors. It enables a so-called real-time monitoring, so that the surgeon can receive feedback on the integrity of the CST and the expected postoperative motor function during tumor resection and consider stopping resection, if necessary, to prevent postoperative deterioration of motor function [2,3]. Intraoperative monitoring can be performed during either awake surgery or general anesthesia. Awake surgery is usually preserved for intraoperative language monitoring. A recent survey conducted in 20 European centers revealed that 58.8% of the patients with brain tumors in eloquent areas were operated under general anesthesia [4]. While subcortical mapping is important to localize the CST during surgery for motor-eloquent brain tumors, monitoring of motor-evoked potentials (MEP) is the only way to assed its integrity and predict postoperative motor function when the surgery is performed under general anesthesia. During resection of brain tumors, MEP can be elicited by either transcranial electrical stimulation (TES) or direct cortical stimulation (DCS) [5]. Each method has its advantages and limitations. The advantages of DCS are the high focality of stimulation, the low stimulation intensity and smaller penetration of the electric field into white matter. The disadvantages are that DCS is not applicable before opening the dura or after closing it, it only allows the assessment of one hemisphere, its inability to insert the strip electrode under the dura in some cases (for example, recurrent tumors due to dural adhesion to the cortex) and possible dislocation of the strip electrode resulting in false positive results and/or time-consuming localization of the primary motor cortex. The advantages of TES-MEP are given by its surgeon-independent application, its availability throughout the surgery and assessment of the unaffected hemisphere for comparison. One of the disadvantages of TES is the possible deep penetration of electric current into the white matter after stimulation with high current intensities and/or using a less focalized electrode combination (for example C4/C3). This might cause a distant activation of the CST and potentially lead to false negative results. In some cases, the surgical approach itself, including skin incision and craniotomy, can interfere with the desired electrode position and, thus, limit the reliability of TES if non-optimal positions of the stimulation electrodes have to be used. This has led to the recommendation of the use of DCS when the motor cortex has to be exposed. For the decision on which method should by applied, neurosurgical units rely on the aforementioned considerations, as well as the individual surgeon’s and neuromonitoring staff’s experience and preferences. Tumor resection under monitoring of MEP has been reported in several studies using DCS [6,7,8] or TES [9,10], with a wide range of sensitivities and specificities for predicting postoperative deficits [6,9,11]. In addition to the different stimulation modalities, there are different alarm criteria to predict postoperative deterioration of motor function [11]. The accuracy of predicting postoperative deficits has a significant impact on clinical outcome, because false negative results (no significant intraoperative MEP change but a postoperative motor deficit) might lead to an unexpected deterioration of motor function, while false positive results (significant intraoperative MEP change without postoperative motor deficit) might lead to an unintended incomplete tumor resection. In addition, the outcome of the operation determines the postoperative quality of life, which has been shown to also affect the patients’ ability to receive adjuvant therapy [12,13]. Because neither the impact of the stimulation modalities nor that of the alarm criteria on postoperative motor function and extent of tumor resection has been investigated yet, we designed the TRANSEKT trial: a multicenter randomized double blind controlled exploratory study which will compare TES-MEP with DCS-MEP for monitoring of MEP in patients undergoing resection of brain tumor under general anesthesia. The aim of this first study is to calculate the required number of patients in a confirmatory trial to test the superiority of one method over the other.

## 2. Methods

### 2.1. Trial Design

This is an interventional, multicenter, double blind, parallel group, exploratory, randomized controlled clinical trial. Eligible patients are randomized to a surgical resection under TES (the intervention arm) or surgical resection under DCS (control intervention).

### 2.2. Objectives

The main aim of the trial is to gain knowledge about the sensitivity and specificity of each transcranial and direct cortical stimulation in prognosticating postoperative deterioration of motor function. This is necessary to calculate the required number of patients in a confirmatory trial to test the superiority of one method over the other. The trial is also designed to study the effect of stimulation modality on the rate of postoperative paresis and extent of tumor resection. A further objective of the trial is to assess functional impairment using Karnofsky performance scale and Barthel index, as well as health-related quality of life using EORTC QOL C30 and the simplified Beck Depression Inventory (BDI-S) in patients undergoing surgery for motor-eloquent brain tumors.

### 2.3. Participating Centers and Recruitment

The following neurosurgical departments intend to participate: University Medical Center Göttingen (UMG), Charité University Clinic, Berlin (CUB), University Medical Center Mainz (UMM), University Medical Center Würzburg (UMW), Klinikum rechts der Isar, School of Medicine, Technische Universität, München (TUM), Ludwig-Maximilians-University, Campus Grosshadern, Munich (LMU) and Inselspital, University Hospital, Bern, Switzerland (UHB). Patients will be recruited for the study from the neurosurgical outpatient clinic or through referral from regional hospitals. The intended recruitment rate will be one to two patients/month per center.

### 2.4. Inclusion Criteria

1. Age ≥ 18 and ≤80 years.
2. Ability to give informed consent.
3. Indication for surgical resection of a supratentorial tumor.
4. Suspected supratentorial glioma or metastasis in close vicinity to the CST confirmed in a preoperative magnetic resonance imaging (MRI).
5. Missing or mild preoperative paresis; Medical Research Council scale for muscle strength (MRC) grades 5 or 4.

### 2.5. Exclusion Criteria

1. Tumor infiltration of the precentral gyrus.
2. Unavailable preoperative MRI.
3. Severe preoperative paralysis (MRC grades 1, 2 or 3).
4. One of the stimulation modalities is not appropriate for intraoperative application according to the neurosurgeon.

### 2.6. Study Timeline

**Figure 1:**
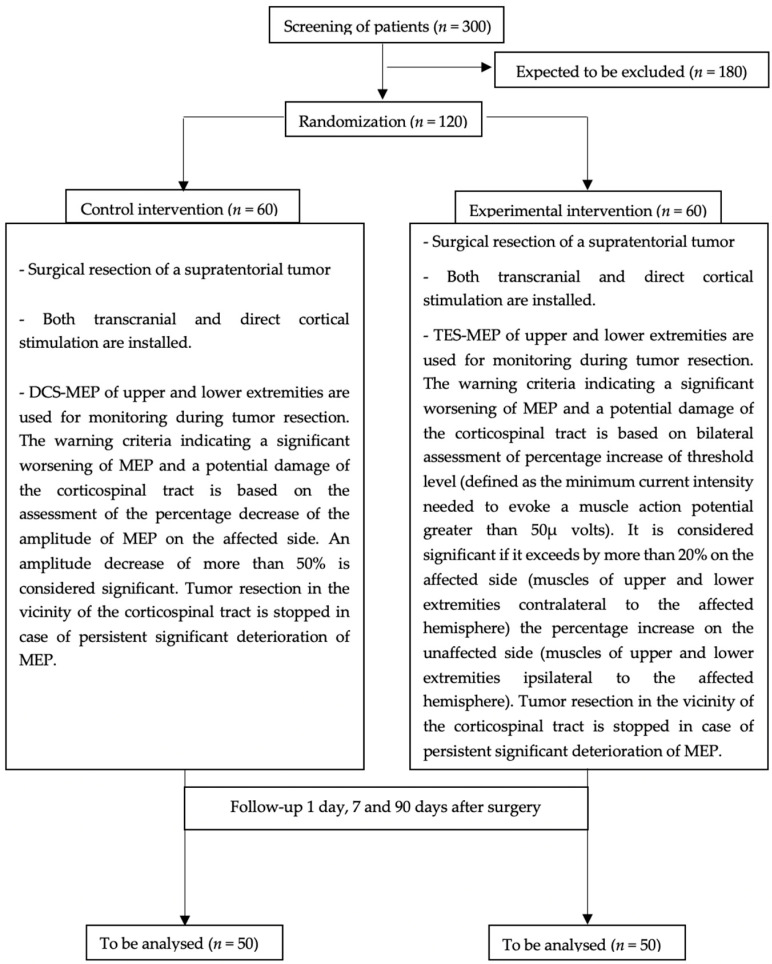
Trial flow diagram. MEP = motor-evoked potentials; TES-MEP = MEP after transcranial electrical stimulation; DCS-MEP = MEP after direct cortical stimulation.

The flow diagram (Figure 1) depicts study interventions and follow-up time points. In sum, evaluations take place by the time at hospital admission upon screening for including the patients in the study, 24 h after surgery, at discharge or 7 days after surgery and at the follow-up visit 3 months postoperatively. The motor status measured by MRC, the Karnofsky performance scale, the Barthel index, the EORTC QOL C30 and the BDI-S will be assessed preoperatively. After these baseline assessments, patients will be allocated randomly to tumor resection under intraoperative monitoring using either the TES or DCS for MEP evaluation. The motor status is assessed 24 h after surgery. The extent of resection will be assessed on postoperative MRI performed within 72 h after surgery. The motor status measured by MRC, Karnofsky performance score and the Barthel index will be assessed at discharge or 7 days after surgery and at follow-up visit 3 months postoperatively, while EORTC QOL C30 and BDI-S will be assessed a second time during a follow-up visit 3 months postoperatively. We expect to complete patient inclusion in twenty-one months. The estimated duration of the study (including follow-up) will be 2 years.

### 2.7. Interventions

#### 2.7.1. Anesthesia

Each participating center should follow its local standard procedure, which applies for all included patients in both arms of the study at that specific center. The following points apply for all participating centers:

- All procedures are performed under general total intravenous anesthesia.
- Muscle relaxant (e.g., rocuronium bromide) is applied for intubation only.
- Anesthesia is induced using propofol and continued using perfusion pumps. Analgesia is applied using sufentanil for the intubation and maintained through remifentanil.
- Use of an infusion pump with TCI function (target-controlled infusion) for maintaining anesthesia and analgesia during surgery is recommended.
- Invasive measurement of blood pressure is performed to maintain stable systolic and mean blood pressure. Body temperature is kept at norm values.
- Monitoring of the depth of anesthesia using EEG Monitoring is recommended.
- Performing regional scalp block or local infiltration at the headframe pins is recommended.

#### 2.7.2. Installation of Intraoperative Monitoring

Installation of intraoperative monitoring starts after intubation and is identical in both study arms. Patients are put in a supine position. Metal pins are used for head fixation, and proximity to the installed electrodes is avoided.

Transcranial electrical stimulation: corkscrew-like electrodes are placed subcutaneously at C1, C2, C3, C4, and Cz according to the international 10–20 electroencephalography system. Skin incision is tailored to avoid interfering with the installed electrodes and, if necessary, sterile electrodes are placed close to the skin incision. The combination C4 to Cz (anode/cathode) is used for stimulation of the left half of the body and C3 to Cz for stimulation of the right half of the body. If a proper stimulation of the lower extremities is not possible with a C3/C4-Cz combination, a C2 to C1 stimulation can be applied for the left leg and C1 to C2 for the right leg.

Direct cortical stimulation: after craniotomy and opening the dura, a strip electrode with four contacts is placed subdurally. Its position is changed until the primary motor cortex can be stimulated through one of the four contacts. Performing phase reversal is allowed on the surgeon’s indication. In addition, presurgical localization of motor cortex and CST using functional imaging or transcranial magnet stimulation can be performed if it is part of the clinical routine at the participating study site [14].

Stimulation parameters: a train of five consecutive pulses with an interstimulus interval of 2–4 ms and an individual pulse width of 0.5 ms pulse duration is applied. The stimulation intensity of DCS is limited to 25 mA according to safety recommendations and the maximum possible stimulation intensity for TES depends on the stimulator output.

Recording of MEP: pairs of subdermal needle electrodes are bilaterally inserted in the following muscles: biceps brachii, extensor digitorum, abductor pollicis brevis, quadriceps femoris, tibialis anterior and abductor hallucis muscles.

#### 2.7.3. Intraoperative Monitoring

A baseline measurement for each muscle is carried out after dural opening with determination of the stimulation threshold for the transcranial MEP or the stimulation threshold and the amplitude for the direct cortical MEPs. During tumor resection, MEP stimulation is performed at least every two minutes for to the randomized method and every 10 min for the other method. The surgeon is informed if a significant MEP deterioration according to the randomized method occurs, and that in case of persistence of the alteration, a significant deterioration of the postoperative deterioration of motor function is to be expected. At the end of the procedure, a final measurement of the stimulation threshold for the transcranial MEP or the stimulation threshold and the amplitude in the direct cortical MEPs is performed.

#### 2.7.4. MEP Deterioration/Alarm Criteria

Transcranial electrical stimulation: the stimulation threshold for each connected muscle is determined separately. It is defined as the lowest current intensity that is necessary to achieve a muscle action potential of minimally 50 μV. The percentage increase in the stimulation threshold from the baseline is calculated for each muscle and each side of the body. The baseline is determined just after opening of the dura.

An increase in the affected side (arm or leg muscles on the opposite side of the affected brain hemisphere) of more than 20% above the percentage increase in the stimulation threshold of the unaffected side of the body (arm or leg muscles on the side of the body ipsilateral to the affected hemisphere) is considered a significant change and warning signal at the same time. In this case, a resection interruption of 10 min in the area of the motor pathways is recommended. Once the MEP has recovered (difference between the two sides below 20%), the resection can be continued. If the deterioration persists, resection termination in the area of the motor pathways is recommended and the risk of postoperative mild/temporary paresis is pointed out.

If the affected side rises by more than 50% above the percentage increase in the stimulation threshold of the unaffected side of the body, the surgeon is informed about the risk of postoperative severe/permanent paresis.

Direct cortical stimulation: the stimulation intensity is set 2 mA above the stimulation threshold and the percentage change in the measured amplitude of the muscle action potential of the affected muscles (arm or leg muscles on the opposite side of the affected brain hemisphere) is calculated.

A reduction in amplitude by 50% compared to the baseline, or an increase in the stimulation threshold by 4 mA (to maintain the amplitude of the baseline) is considered a significant change and a warning signal at the same time. A resection interruption of 10 min in the area of the motor pathways is recommended. Once the amplitude has recovered (reduction of less than 50% or increase in the stimulation threshold below 4 mA), the resection can be continued. If the deterioration persists, resection termination in the area of the motor pathways is recommended and the risk of postoperative rather slight/temporary paresis is pointed out.

In case of amplitude loss under a maximum stimulation intensity of 25 mA, the surgeon is informed about the risk of postoperative severe/permanent paresis.

### 2.8. Outcomes

#### 2.8.1. Primary Outcome Measure

Status of motor function at discharge or one week following surgery is the primary outcome measure. It will be assessed using MRC. The change of motor status will be categorized in two groups, improvement or no change, and deterioration of one MRC grade or more.

#### 2.8.2. Main Secondary Outcome Measure

Proportion of remaining tumor will be measured based on pre- and postoperative MRI. Preoperative MRI is performed within 1–2 weeks prior to surgery and postoperative MRT is performed within 72 h after surgery. Thin slice sagittal magnetic resonance T1 contrast imaging in case of enhancing tumors and T2 flair images (minimum 160 slices) are transferred into iPlan 3.0 cranial (BrainLab, Munich, Germany). This software enables a semiautomatic object drawing using the object creation tool. To measure the tumor volume, its area is encircled in each slice with manual correction if required. Afterwards, the tumor volume expressed in ccm, is extracted using the advanced manipulation function. The measurement is performed independently by a neurosurgeon and a neuroradiologist.

### 2.9. Methods against Bias

Selection bias is minimized by randomizing patients, stratified by center, tumor localization and contrast medium enhancement (1:1 treatment ratio). Block randomization with random block length will be performed. Performance bias is reduced by the double-blind study design. Neither the patient nor the neurosurgeon will be aware of the applied warning criteria. Detection bias is reduced by blinding outcome assessors to the data until the end of the study. A statistical analysis plan (SAP) will be written prior to database lock and all results will be published independently of specific findings to minimize reporting bias.

### 2.10. Blinding

Neither the patient nor the neurosurgeon will be aware of the applied monitoring method during tumor resection. If randomized to TES, detection bias resulting from a possible dislocation of the strip electrode will be minimized through regular impedance measurement (every 2 min) and through performing DCS every 10 min. The medical technical assistant who performs the intraoperative monitoring is blinded to the motor outcome and those who assess the motor outcome are blinded to the intraoperative monitoring.

### 2.11. Sample Size

For the calculation of the sample size, it is assumed that both the sensitivity and specificity of transcranial stimulation is 99% [9,10], and a sensitivity of 80% and specificity of 95% for direct cortical stimulation [6,7]. Furthermore, a prevalence of 30% for the occurrence of motor deficits on discharge or 7 days after surgery is assumed. With 110 patients, a 95% confidence interval for the difference in sensitivities would be 29% percentage points wide and 95% confidence interval for the difference in specificities is 13% percentage points wide. This level of accuracy is sufficient for planning a confirmatory study. With a dropout rate of 10%, the aim is to recruit 120 patients. The number of cases was calculated in R 3.6.0.

### 2.12. Definition of Population

The primary analysis is carried out on the per-protocol set (PP) as usual in non-inferiority studies. Sensitivity analyses with the intention-to-treat (ITT) population and the as-treated (AT) population will be carried out and any differences will be discussed.

### 2.13. Statistical Analysis

A statistical analysis plan with more technical details on the methods used will be written prior to database lock.

#### 2.13.1. Primary Endpoint

The sensitivity and specificity of the transcranial and direct cortical stimulation with regard to motor deficit at discharge or 7 days after the operation are calculated and compared between the procedures using the Farrington-Manning test. Sensitivity refers to the probability of having a motor deficit at discharge (or 7 days after surgery), after reaching a predefined deterioration of MEP according to the applied alarm criterion during tumor resection. Specificity refers to the probability of not having a motor deficit at discharge (or 7 days after surgery) while not reaching a predefined deterioration of MEP according to the applied alarm criterion during tumor resection. The sensitivity is tested for superiority to a two-sided significance level of 5%; the specificity is tested for non-inferiority with a non-inferiority threshold of 10% with a one-sided significance level of 2.5%. One method is superior to another if it has been possible to demonstrate superiority in sensitivity and non-inferiority in specificity (intersection hypothesis). A correction of control of the type I error is, therefore, not necessary. Despite the conservative design, the main aim of the study is to gain a first understanding of the underlying differences in prognostic sensitivity and specificity between the two procedures.

#### 2.13.2. Secondary Endpoints

The secondary endpoint, extent of resection, is analyzed between the randomization groups using an ANCOVA (Analysis of Covariance) with baseline tumor mass as a covariate. The proportion of patients with and without motor deficit is compared between the randomization groups using the chi-square test. Differences in the Barthel Index and in the Karnofsky Performance Score at discharge and after 3 months as well as in EORTC QOL C30 and BDI-S after 3 months are analyzed using an ANCOVA.

### 2.14. Study Initiation

An on-site study initiation will be undertaken at each participating center. Details of the interventions including TES and DCS will be discussed thoroughly and demonstrated in practice using a standardized scenario that will be run on the device that is used for intraoperative monitoring. In addition, each participating study center receives a study-specific training on good clinical practice requirements.

### 2.15. Study Monitoring

The Clinical Trial Unit of the University medical center Göttingen applies a risk-based monitoring concept, which is described in a study-specific monitoring plan. The purpose of trial monitoring is to verify that:

1. The rights and well-being of patients are protected.
2. The reported trial data are accurate, complete, and verifiable from source documents.
3. The conduct of the trial is in compliance with the currently approved protocol/amendment(s), with GCP, and with the applicable regulatory requirement(s).

### 2.16. Adverse Events

The applied methods for intraoperative monitoring are already well established in the clinical routine. We expect study-related adverse events to take place perioperatively at a very low rate and include epileptic seizures and lip and tongue injuries. These will be considered in the results and final analyses. In case of intraoperative seizures, all kinds of stimulations are stopped, and cold saline is applied on the exposed cortex along with intravenous application of Benzodiazepine or Thiopental if needed.

An interim analysis will take place after inclusion of 60 patients. It will evaluate the rate of postoperative paresis and the extent of tumor resection in comparison to the available literature.

### 2.17. Drop-Out Criteria and Termination of the Study

Withdrawal of patient’s consent, significant violation of the study protocol, cerebral ischemia, or intracerebral hemorrhage that occurs in the postoperative course and requires either a surgical intervention or an ICU-surveillance and is associated with a deterioration of the motor status are the main drop-out criteria. A violation of the study protocol is considered significant if it affects blinding or the interpretation of intraoperative monitoring.

The primary investigator will terminate the study when the interim analysis reveals a significantly higher rate of postoperative paresis or a lower extent of tumor resection than what has been reported in the literature. Major unexpected events can also lead to the termination of the study.

### 2.18. Data Management

Data will be entered remotely via an electronic case report form (eCRF) and stored in a provided SecuTrial^®^ database. SecuTrial^®^ is an established web solution facilitating the development of 21 CFR Part 11 conform web applications. Offsite data checks for plausibility and missing data will be performed during the complete course of the study to ensure high data quality and monitor recruitment. Inconsistent data will result in queries and/or planned visits for source data verification. After database deactivation, a copy of the database is password encrypted and archived for a 10-year period within the UMG. After publication of the primary results, all data/metadata will be anonymized and published in an open access repository (e.g., Publisso) in order to guarantee data access by third parties. To obtain long-term outcomes, including progression-free survival and overall survival, we plan to transfer the database to a prospective registry in a separate project.

## 3. Discussion

The presented trial is the first to address intraoperative neuromonitoring with TES and DCS-MEP during brain tumor resection under general anesthesia in a prospective randomized manner. Through its blinded design, the study is expected to demonstrate the true effect of each of the investigated monitoring methods on the motor status as a primary endpoint and on the extent of tumor resection as a secondary endpoint. There are several reports on intraoperative monitoring under general anesthesia with a large diversity regarding the stimulation modalities and the alarm criteria [15,16,17,18,19]. In general, one of two stimulation modalities are used to gain MEP, i.e., TES or DCS [6,20,21,22,23]. The reason to choose one method over the other has rarely been discussed. The inclusion criteria of the current trial define tumor localization that allows for an appropriate use of both methods. Patients with tumor infiltration of the precentral gyrus will not be included, because the craniotomy in these patients has to expose the primary motor cortex and, thus, prohibits a correct installation of the dermal electrodes needed for TES. Patients with severe preoperative paresis will also not be included, as this might be associated with an unsuccessful eliciting of intraoperative MEP.

Interpretation of changes in intraoperative MEP can be challenging. Available alarm criteria aim at preventing and predicting deterioration of postoperative motor function. An alarm criterion based on bilateral evaluation of motor threshold has been recently introduced for the evaluation of transcranial MEP during resection of supratentorial tumors [9]. In comparison to the conventional amplitude-based alarm criterion, the threshold criterion had a higher sensitivity and specificity in prediction of postoperative deterioration of motor function [10]. Therefore, it was chosen to be used in the study arm that applies TES during tumor resection. For DCS, both threshold- and amplitude-based alarm criteria were described with high sensitivity and specificity, and there is no study that compares these criteria during DCS. Therefore, a combination of both criteria was chosen to be used in the study arm that applies DCS during tumor resection.

The primary endpoint was set at discharge or one week after surgery, because this is the time point at which patient ability to undergo adjuvant therapy is evaluated. Motor status at the 3-month follow-up was not chosen as a primary but as a secondary endpoint, because many patients with brain tumors have to receive radio- and/or chemotherapy prior to this follow-up, which might be associated with a secondary deterioration of motor function, leading to evaluation bias. Using the Barthel index and Karnofsky index as secondary endpoints is necessary from our point of view, because both are strongly correlated to the motor function and used for evaluation of patient’s ability for further treatment or the need for rehabilitation or nursing facilities. All primary and secondary endpoints, including Barthel and Karnofsky indexes, are very significant measures for the quality of life and the ability to undergo treatment and influence the prognosis of patients with brain tumors. They have been validated and utilized as primary or secondary endpoint in clinical trials [24,25,26]. Self-reported quality of life using EORTC QOL C30 and BDI-S will enable us to gain concrete information about the effect of deterioration of motor function on the patient’s quality of life and their emotional health. These questionnaires have been validated in numerous papers addressing quality of life in cancer patients [27,28,29,30,31].

## 4. Conclusions

This trial was designed as an exploratory study to investigate sensitivity and specificity of TES and DCS in predicting postoperative motor deficits and study the influence of stimulation modality on the extent of tumor resection. The results of this trial will allow us to calculate the required number of patients in a confirmatory trial to test the superiority of one method over the other and will probably pave the way for a standardized evidence-based use of intraoperative monitoring during resection of supratentorial tumors. This will benefit patients suffering from brain tumors as well as neurosurgeon and neurophysiologist that are involved in the surgical treatment of brain tumors.

## Data Availability

This manuscript is based on the most recent protocol version, version 3.0 October 2020.

## Trial Status

The first patient was included on 3 December 2020. Currently, three German study sites are active. The activation of the remaining study sites is under preparation.
